# Direct versus conventional sonication for the diagnosis of joint infection: A comparative study

**DOI:** 10.6026/973206300213321

**Published:** 2025-09-30

**Authors:** Zahid Bashir, Heena Dixit Tiwari, Fazeena Karimalakuzhiyil Alikutty, Rahul Tiwari, Deepak Sharma, Anil Managutti, Afroz Kalmee Syed

**Affiliations:** 1 Department of Orthopedics, Ortho One Hospital, Coimbatore, Tamil Nadu, India; 2 Department of Medical Health Administration, Index Institute, Malwanchal University, Index City, Nemawar Road, Indore, Madhya Pradesh, India; 3 Department of Preventive Dental Sciences, Ibn Sina National College for Medical Studies, Jeddah, Saudi Arabia; 4 Department of Oral and Maxillofacial Surgery, Narsinhbhai Patel Dental College and Hospital, Sankalchand Patel University, Visnagar, Gujarat, India; 5 Department of Oral and Maxillofacial Pathology, Scientific Medical Writer, Writing and Publications, Tenali, Andhra Pradesh, India

**Keywords:** Periprosthetic joint infection, sonication, blood culture, biofilms, time-to-positivity

## Abstract

Periprosthetic joint infection (PJI) remains a major cause of revision arthroplasty, and accurate diagnostic methods are essential. In this comparative study of 196 revision cases (94 direct vs. 102 conventional sonication), 116 (59.2%) met criteria for PJI. Direct sonication achieved higher sensitivity (86.2%, 95% CI 78.3-92.0) compared with conventional sonication (70.7%, 95% CI 60.4-79.5; p < 0.01), while specificity was comparable (90.3% vs. 94.4%). Importantly, the time-to-positivity (TTP) was significantly shorter with direct sonication (median 2.1 days vs. 3.0 days; p = 0.002), particularly for Gram-positive organisms. These findings demonstrate that direct sonication enhances diagnostic yield, accelerates pathogen recovery, and supports its integration into standardized PJI diagnostic pathways for earlier targeted therapy.

## Background:

Periprosthetic joint infection (PJI) is one of the most serious complications following arthroplasty, often leading to revision surgery, prolonged hospitalization and higher costs [1]. Diagnosis remains challenging because biofilm-forming bacteria adhere to prosthetic surfaces and are difficult to detect using standard tissue or synovial fluid cultures [2]. Sonication of explanted prostheses has emerged as an important adjunct technique to improve diagnostic yield. By dislodging biofilm-associated microorganisms, sonicate fluid culture (SFC) demonstrates higher sensitivity compared with conventional culture methods [3]. Recent reviews and multicenter studies have confirmed that sonication improves detection in low-grade or partially treated infections [4, 5]. Technological advances now allow direct intraoperative sonication, where ultrasonic probes are applied during surgery with immediate inoculation into blood culture bottles. This approach shortens specimen handling time and improves recovery of fastidious organisms [6]. Zouitni *et al.* showed that extended incubation improved detection of low-virulence organisms in PJI [1]. Baez *et al.* reported longer time-to-positivity and higher fungal recovery with 14-day cultures compared to 5-day cultures [8]. Other methodological refinements, such as using cut off thresholds (e.g., ≥20 CFU/10 mL) and inoculating into automated culture systems, have further improved diagnostic performance [9, 10]. Therefore, it is of interest to compare the diagnostic performance of direct versus conventional sonication in PJI, focusing on sensitivity, specificity and time to positivity.

## Materials and Methods:

This retrospective comparative cohort study was conducted at a tertiary referral center and included consecutive hip and knee revision arthroplasties performed between January 2022 and June 2025. Cases were classified as 'infection unlikely,' 'infection likely,' or 'infection confirmed' based on EBJIS-aligned diagnostic criteria. Patients underwent preoperative serum and synovial testing per institutional protocol. Exclusion criteria were missing culture data, re-revision within 14 days for non-infectious indications, or incomplete sonication workflow. Direct sonication: Explanted implants were processed intraoperatively using a standardized vortex-sonicate-vortex cycle, with concentrate directly inoculated into aerobic and anaerobic blood culture bottles under sterile conditions. Conventional sonication: Specimens were transported to the laboratory in Ringer's solution and processed using the same vortex-sonicate-vortex protocol, followed by plating to solid media. Primary outcomes included sensitivity, specificity and time-to-positivity (TTP) compared with final clinical diagnosis adjudicated by multidisciplinary review. Secondary outcomes included contamination, polymicrobial detection and concordance with tissue culture. Statistical analysis used χ^2^ or Fisher's exact tests for proportions and Mann-Whitney U for continuous variables. The study received ethics approval from the institutional review board and informed consent was waived due to its retrospective nature.

## Results:

A total of 196 revision arthroplasties were analyzed: 94 underwent direct sonication and 102 conventional sonication. Of these, 116 cases fulfilled criteria for PJI. Baseline demographics and preoperative markers were comparable between groups (Table 1 - see PDF). Direct sonication demonstrated higher sensitivity (86.2%, 95% CI 78.3-92.0) compared with conventional sonication (70.7%, 95% CI 60.4-79.5). Specificity was similar between groups (90.3% vs 94.4%). Time-to-positivity was significantly shorter with direct sonication (median 2.1 days vs 3.0 days, p=0.002), particularly for Gram-positive organisms. Direct sonication increased detection of low-virulence organisms and yielded more polymicrobial results, while contamination rates remained low and comparable. Detailed diagnostic outcomes are shown in Table 2 (see PDF).

## Discussion:

This comparative study shows that intraoperative direct sonication achieved superior diagnostic sensitivity and reduced time-to-positivity compared with conventional laboratory sonication, without a significant loss of specificity. Our findings align with recent clinical studies that reported similar improvements in yield and TTP with direct workflows [11]. Enhanced detection of low-virulence organisms and improved identification of polymicrobial infections were additional advantages [12]. Direct sonication provides an operational benefit by reducing transport delays and inoculating into blood culture bottles immediately, which accelerates microbial recovery. Faster TTP allows earlier pathogen-directed therapy, supporting antimicrobial stewardship [13]. These advantages are especially relevant in chronic or partially treated infections, where conventional methods often underperform [14]. However, limitations exist. Contamination risk remains a concern, though rates were low in this study. Generalizability may be limited by the single-center design and specific laboratory practices [15]. Optimal cut offs for colony-forming units and interpretation in low-likelihood cases require further validation [16]. Additionally, the retrospective design may introduce bias despite multidisciplinary case adjudication [17]. Future multicenter prospective trials are needed to confirm reproducibility, refine thresholds and standardize workflows [18]. Integration of molecular diagnostics and metagenomic approaches with direct sonication could further improve sensitivity [19]. Overall, the findings support direct sonication as a practical and effective adjunct to existing diagnostic algorithms for PJI [20].

## Conclusion:

Intraoperative direct sonication increased diagnostic sensitivity and shortened TTP compared with conventional sonication in PJI workups. Integrating direct sonication into EBJIS-aligned pathways may accelerate organism identification and inform timely therapy. Prospective multicenter studies should refine selection criteria, contamination safeguards and cost-effectiveness endpoints.
